# Anion‐Driven Surface Reconstruction Enables Direct Regeneration of LiCoO_2_ Cathode Material

**DOI:** 10.1002/advs.77656

**Published:** 2026-09-27

**Authors:** Mengting Zheng, Shangshu Qian, Meng Li, Shanqing Zhang, Jun Lu

**Affiliations:** ^1^ College of Chemical and Biological Engineering Zhejiang University Hangzhou China; ^2^ Centre For Clean Environment and Energy School of Environment and Science Gold Coast Campus Griffith University Gold Coast Australia; ^3^ Institute For Sustainable Transformation School of Chemical Engineering and Light Industry Guangdong University of Technology Guangzhou China

**Keywords:** direct recycling, eutectic molten salt regeneration, high voltage cathode, layered cathode, spent LiCoO_2_

## Abstract

The escalating volume of decommissioned lithium cobalt oxide (LCO) batteries necessitates recycling strategies capable of adding value by transforming spent cathodes into high‐performance materials. While eutectic molten salt systems have emerged as a promising liquid–solid medium for direct regeneration, the selection of salts has been largely empirical, and the critical role of anions in governing the relithiation efficacy and interface chemistry remains underappreciated. Herein, we demonstrate that the strategic choice of anions in a binary molten salt flux is paramount to achieving structural repair and performance enhancement. We identify that anions with high adsorption energy to the CoO_6_ slabs and low dissociation energy for Li^+^, such as Cl^−^, are optimal. This anion‐mediated process directs a preferential crystallographic reconstruction along the *c*‐axis and fosters the development of a Li−O enriched, (104)‐facetted surface. Consequently, the upcycled LCO cathode retained over 95% of its original capacity after 1000 cycles at 4.2 V. Even at an elevated voltage of 4.6 V, the upcycled LCO retains 85.5% of its capacity after 100 cycles at 0.5 C. This work underscores that understanding and engineering the anion in molten salts could be a powerful step toward the rational design of advanced upcycling protocols.

## Graphical abstract

The anion scpecies in molten salt systems critically govern the surface reconstruction of regenerated LiCoO_2_. LiOH−LiCl flux, featuring strong Cl^−^ adsorption and facile dissociation, directs preferential relithiation on (003) facets and forms a Li−O enriched interface. This anion‐mediated reconstruction effectively restores the surface structure and merits much‐enhanced electrochemical performance.

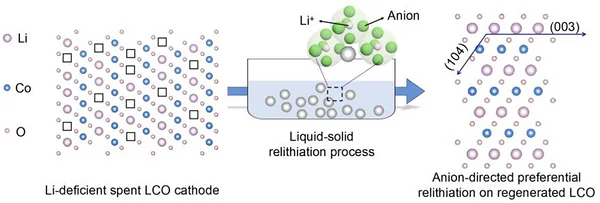

## 1 Introduction

Li‐ion batteries (LIBs) have become indispensable in modern society, powering everything from portable electronics to electric vehicles. Among various cathodes, lithium cobalt oxide (LCO) is prized for its high energy density and safety, making it a ubiquitous choice [1]. Extensive efforts have been devoted to pushing the practical capacity of LCO by operating at elevated voltages; however, this approach stresses its layered structure, triggering adverse phase transitions and compromising stability [2, 3]. Consequently, commercial LCO is typically limited to a cut‐off voltage of 4.2 V, utilizing only about half of its theoretical capacity. This underutilization, coupled with the massive scale of production, leads to a growing stream of spent batteries. The valuable metals within also pose a significantly environmentally toxicity risk if not managed properly [4, 5].

From a sustainable perspective, recycling decommissioned LCO cathode could offer a unique opportunity not just for recovery, but for upcycling, i.e., transforming spent materials into cathodes with performance surpassing that of their commercial counterparts. Unfortunately, conventional metallurgy‐based recycling processes, including pyro‐ and hydro‐metallurgy, are ill‐suited for this ultimate goal. These energy‐intensive methods break down the electroactive materials into their constituent elements, consuming large amount of energy and generating significant waste [6]. Such a downcycling strategy introduces substantial chemical complexity, often rendering subsequent separation and purification economically unviable [7].

Emerging direct regeneration strategies that add value without shredding the cathode structure present a more promising path [8]. These can be categorized into solid–solid [9, 10, 11] and liquid–solid reaction pathways [12, 13, 14]. The key difference lies in the form of the lithium (Li) source used to compensate for Li loss. Solid–solid processes, i.e., calcination with solid‐state Li_2_CO_3_ or LiOH, often suffer from insufficient contact between the solid Li source and the spent cathode particles [15]. In contrast, liquid–solid pathways, such as hydrothermal [16] or molten salt treatments [17, 18], employ liquid‐phase Li sources, enabling more efficient and effective relithiation.

Eutectic Li‐based molten salt systems are particularly attractive as they facilitate structure repair and modification at lower temperatures. By combining two or more salts, the melting point of the mixture is dramatically reduced compared to a single salt. More importantly, the local environment of the Li^+^ in a eutectic mix is complex and distinct from a single salt; anions coordinate with Li^+^ to form various complex ions to achieve charge balance. We posit that the “de‐coordination” of these Li‐anion complexes is a critical step, and the participating anions therefore play a vital, yet overlooked, role in governing relithiation dynamics. While several eutectic systems such as LiOH−Li_2_CO_3_ [13] LiI−LiOH [19], KCl−LiNO_3_ [20], and LiOH−KOH−Li_2_CO_3_ [21] have been empirically shown to recycle or upcycle layered cathode materials, the current selection of molten salt remains largely a trial‐and‐error process. The fundamental effects of different anions on the relithiation dynamics and the resulting interface chemistry of the recycled materials are poorly understood, hindering the rational design of advanced upcycling processes.

Herein, we theoretically and experimentally decipher the working mechanism of liquid–solid regeneration to help establish selection criteria for effective anions. The surface Li‐deficiency features of spent cathode particles indicate that anions which strongly adsorb onto the CoO_6_ slabs adjacent to Li vacancies, yet allow for facile dissociation of Li^+^ are highly desirable for maximizing relithiation. Guided by this principle and DFT calculation results, we employed a binary eutectic salts of LiOH and LiCl, to upcycle spent LCO. The specific interaction of Li^+^−Cl^−^ with the lithium deficiency surface of LCO promotes a preferential crystallographic reconstruction along the *c*‐axis, yielding an ultrahigh I(003)/I(104) ratio of 4.38. Furthermore, the efficient Li uptake facilitates the formation of Li−O enriched grains and a (104)‐enclosed interface. A surprising cycling performance at 4.2 V was realized, delivering about 124.4 mAh/g and reserving over 95% of the original capacity even after 1000 cycles. Moreover, using conventional carbonate electrolyte, the upcycled cathode demonstrates a capacity retention of 85.5% at 0.5 C within 3.0−4.6 V after 100 cycles. This work provides fundamental insight into how anions in a Li^+^ flux govern microstructure evolution, paving the way for the rational design of molten salts for the directional upcycling of layered cathode materials.

## 2 Results and Discussion

### 2.1 Rationale for Anion Selection in Molten Salt Relithiation

Liquid‐state molten salts at elevated temperatures that feature high ionic concentration and good homogeneity have been demonstrated as an effective Li compensation medium [22]. Unlike other liquid−solid methods that require high‐pressure conditions, molten‐salt‐based reaction media leverage the concentration gradient and the interaction between the components of the reaction media to enable Li compensation and facilitate the reconstruction of crystal structures at ambient pressure. Given the dynamic and unique local configurations of Li^+^ ions in molten salt systems, Li^+^ ions are normally coordinated by anions with a coordination number ranging from 4 to 5, depending on the types of anions [23, 24]. Given the unique short‐range‐order structure of Li molten salt, the coordinated Li^+^ moves and interacts with the defective cathode materials to realize relithiation. In this case, the types of coordinated anions are considered to exhibit much‐underestimated effects on the microstructure of the recycled or upcycled materials, which are demonstrated in this work by the differences between the microstructures of recycled LCO cathode from different molten salt systems.

**FIGURE 1:**
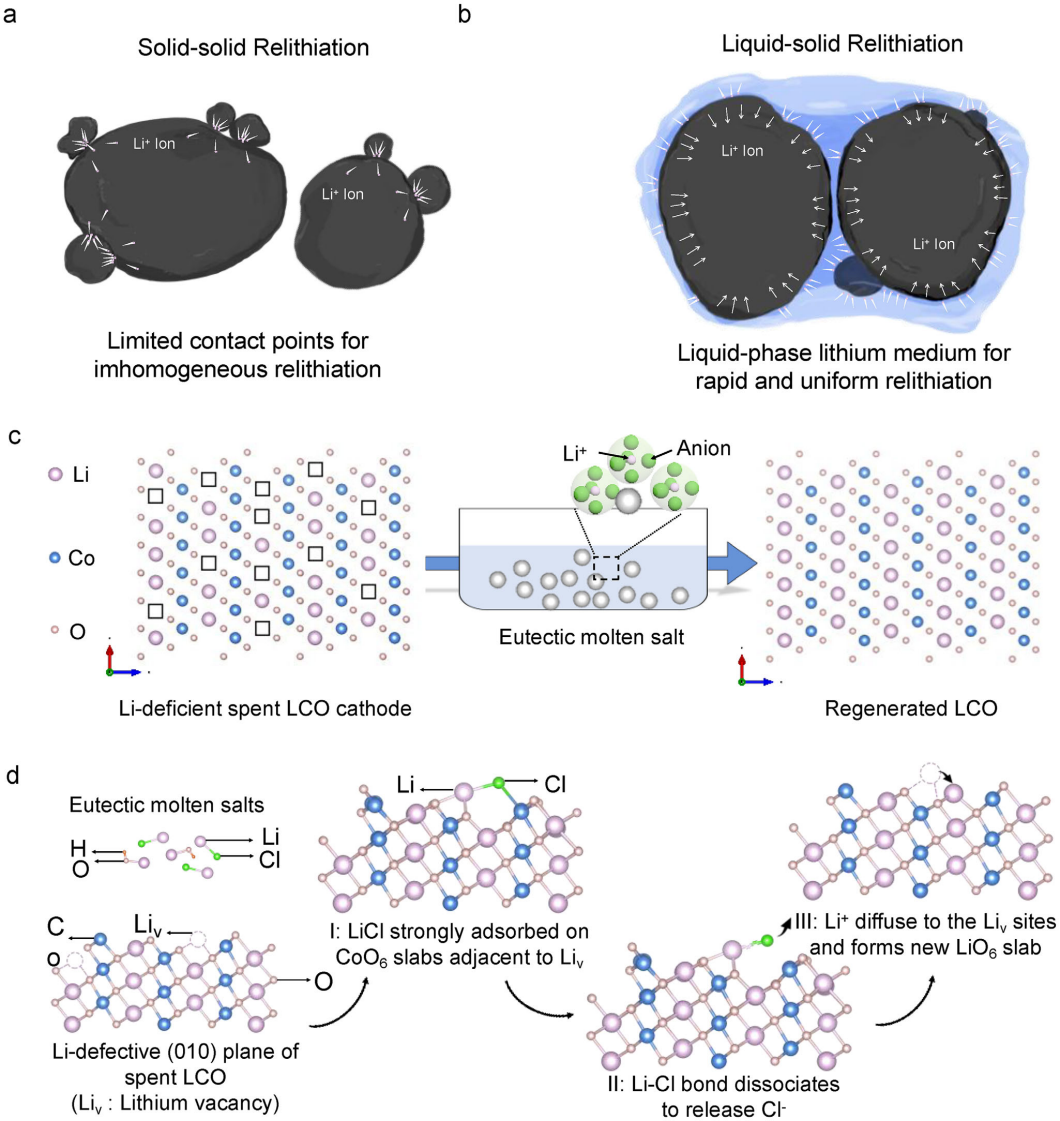
Schematic illustrations of the relithiation mechanisms. Schematic diagrams of (a) solid−solid relithiation and (b) liquid−solid relithiation pathways. (c) Schematic illustration of the lithiation process of defective LCO cathode in the presence of binary eutectic LiCl−LiOH molten salts. (d) Illustration of the proposed mechanism for lithium replenishment in a deficient LCO cathode within a molten eutectic medium, involving the adsorption, dissociation, and diffusion of Li^+^ ions.

Our approach to direct upcycling hinges on the rational selection of anions in the eutectic molten salt system to reconstruct the surface structure. Conventional solid‐state relithiation suffers from poor solid−solid contact and slow kinetics (Figure 1a). Before the melting of supplementary lithium salts at their melting point, Li^+^ ions diffuse only along the contact surfaces between solids (shown as the white arrow in Figure 1a), resulting in inefficient Ostwald ripening [15]. Limited lithiation at the contact point often fails to sustain regeneration efficiency. In a liquid molten salt flux, Li‐deficiency LCO particles are exposed completely to the ionically conductive medium, i.e., molten Li salt mixtures that offer complete and uniform contact between deficiency surface and the lithiation media (Figure 1b,c). In the dynamic ionic environment of a eutectic molten salt, we posit that the relithiation of degraded LCO is governed by a three‐step process (Figure 1d): (i) the surface‐specific adsorption of Li‐anion complexes, (ii) the dissociation of the Li^+^ ion from its anionic coordination shell, and (iii) the subsequent diffusion of the liberated Li^+^ into the bulk lattice. The local coordination structure of the Li^+^ flux, defined by the anion chemistry, is critically important. Different anions create distinct coordination environments with varying binding strengths, which directly dictate the thermodynamics and kinetics of the first two steps.

To validate this hypothesis at the atomic level, Density Functional Theory (DFT) calculations were conducted on a Li‐defective LCO model with an exposed (010) plane (Figure S1). Two binary molten salts, LiOH‐LiCl and LiOH‐LiNO_3_, were selected to figure out the role of different anions and the results confirm a decisive anion effect. The calculation reveals that the coordinated Li^+^ ions primarily interact with the lattice oxygen in CoO_6_ octahedra around the Li defects, which might be due to the electrostatic repulsion between the lattice Li and the Li^+^ ions and the electronegative nature of lattice oxygen. In the presence of LiCl, Li^+^ forms three Li─O bonds involving the lattice oxygen in the CoO_6_ octahedron, and Cl^−^ bonds with two lattice oxygen from another adjacent octahedron, leading to an adsorption energy of −3.10 eV (Figure S2). As for the system with LiNO_3_, only two Li─O bonds and one Co─O bond could be formed between (010) and NO_3_^−^ during the adsorption process with a much lower adsorption energy of −1.52 eV. Furthermore, the dissociation energy for Li^+^ from Cl^−^ is markedly lower than from NO_3_^−^ (Figure S2). This combination is ideal: strong adsorption efficiently anchors the lithium source to the active defect site, while the low dissociation barrier facilitates the rapid release of “free” Li^+^ ions [25]. This mechanistic insight, corroborated by studies on Li^+^ mobility in molten salts where Li^+^ is often the most mobile species, establishes Cl^−^ as an ideal “carrier anion” for relithiation.

### 2.2 Degraded Layered Structure at the Near Surface Region

**FIGURE 2:**
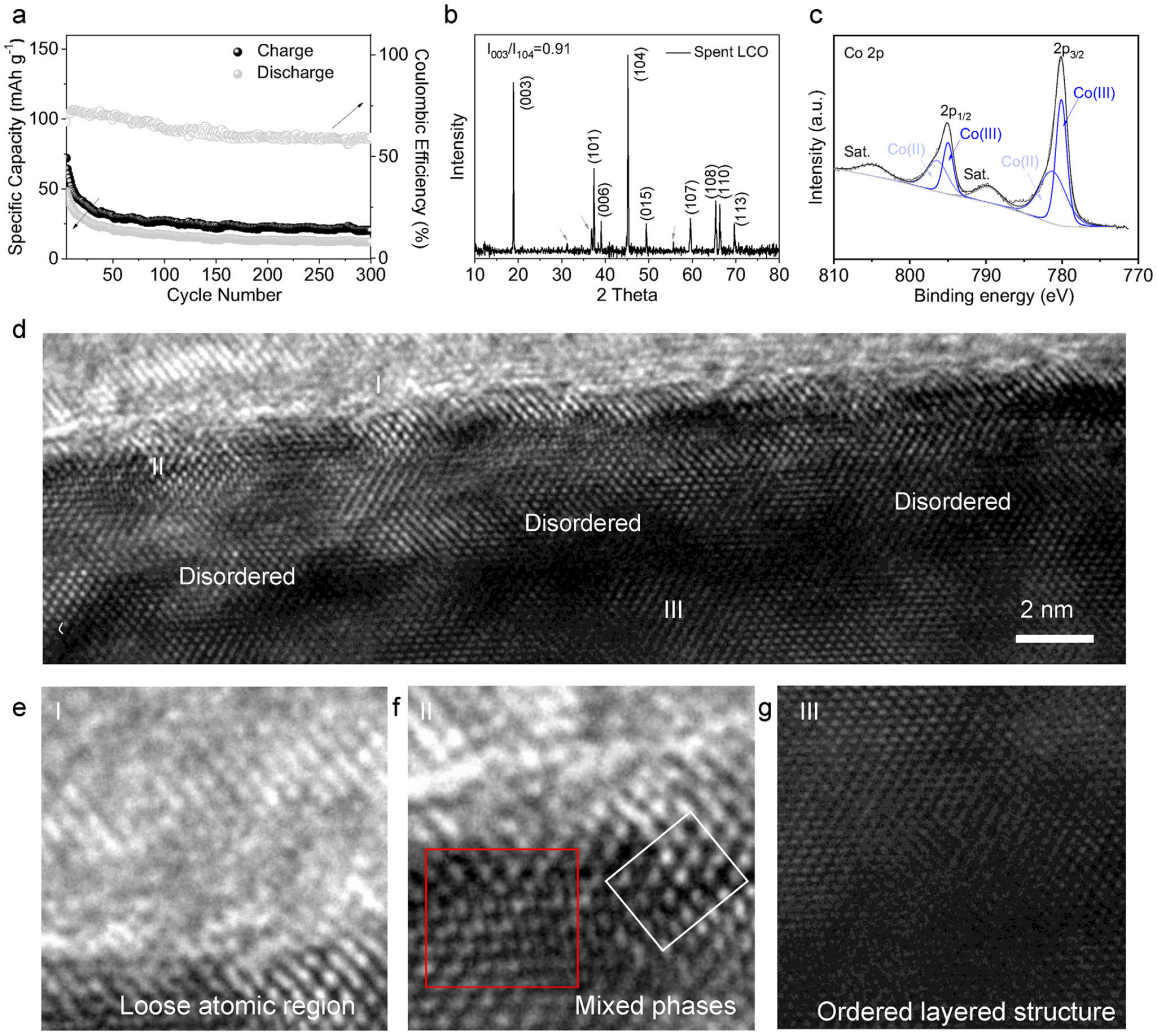
Degraded layered structure at near surface region of spent LCO cathode. (a) The decay cycling performance of spent LCO. (b) XRD pattern of spent LCO. (c) High‐resolution Co 2p XPS spectra of spent LCO. (d) TEM image of the near‐surface area of spent LCO particle. (e–g) Magnified views of the regions labeled I, II, and III in d, highlighting specific structural features.

To establish a baseline for upcycling, we first systematically characterized the spent LCO cathode harvested from waste commercial prismatic cells (Figure S3 and Note S1). The inferior electrochemical performance of spent LCO is immediately apparent, exhibiting significant capacity loss and poor cycling stability at 0.2 C within a voltage range of 3.0–4.2 V (Figure 2a). To unravel the origin of this performance decay, we employed a multi‐faceted structural analysis. X‐ray diffraction (XRD) reveals that the foundational layered α‐NaFeO_2_ structure (*R*‐*3m* space group) is largely preserved in the bulk (Figure 2b) [26]. However, the presence of additional peaks corresponding to a spinel‐like Co_3_O_4_ phase indicates localized structural collapse due to extensive lithium loss during cycling. This bulk‐level cation disorder is further quantified by the intensity ratio of the (003) to (104) peaks (I(003)/I(104)) [27]. A value of 0.91, well below the 1.2 threshold for a well‐ordered layered structure, confirms significant degradation. To understand the chemical state at the surface, we turned to X‐ray photoelectron spectroscopy (XPS). The high‐resolution Co 2p spectrum (Figure 2c) deconvolutes into characteristic peaks for Co^3+^ and a substantial contribution from Co^2+^, with the latter accounting for over 70% of the spectral area. This prevalence of low‐valence Co^2+^ aligns with the formation of Co_3_O_4_ identified by XRD and directly points to the reduction of Co^3+^ consequent to Li^+^ extraction [28].

To spatially resolve this degradation, we conducted transmission electron microscopy (TEM). Low‐magnification TEM (Figure 2d) confirms that the structural damage is not uniform but progresses from the surface inward [29]. Analysis of the outermost surface (Region I, Figure 2e) shows a severely degraded and loosened atomic arrangement, while the near‐surface region (Region II, Figure 2f) exhibits a clear phase transformation to a spinel structure, as confirmed by Fast‐Fourier Transform (FFT) patterns (Figure S4). In stark contrast, the bulk region (Region III, Figure 2g) retains a well‐ordered layered structure. This microstructural evidence paints a clear picture: the bulk crystal framework remains largely intact, but the particle suffers from a surface‐to‐core gradient of degradation, characterized by Li deficiency, a spinel phase transformation, and a dominant Co^2+^ presence at the surface. It is this very degraded interface, which is responsible for irreversible reactions and impeded ion transport, that dictates the inferior electrochemical performance, positioning it as the critical target for a surface‐specific Li‐resupply approach. This surface‐specific Li‐deficient feature provides a clear rationale for direct relithiation. However, the success of such strategies, particularly in molten salt systems, depends profoundly on the yet‐to‐be‐explored interactions between the lithium flux and the degraded surface. Investigating these interactions is thus key to unlocking controlled and efficient upcycling.

### 2.3 Relithiation With Distinct Li^+^ Ion Reinsertion Behavior

**FIGURE 3:**
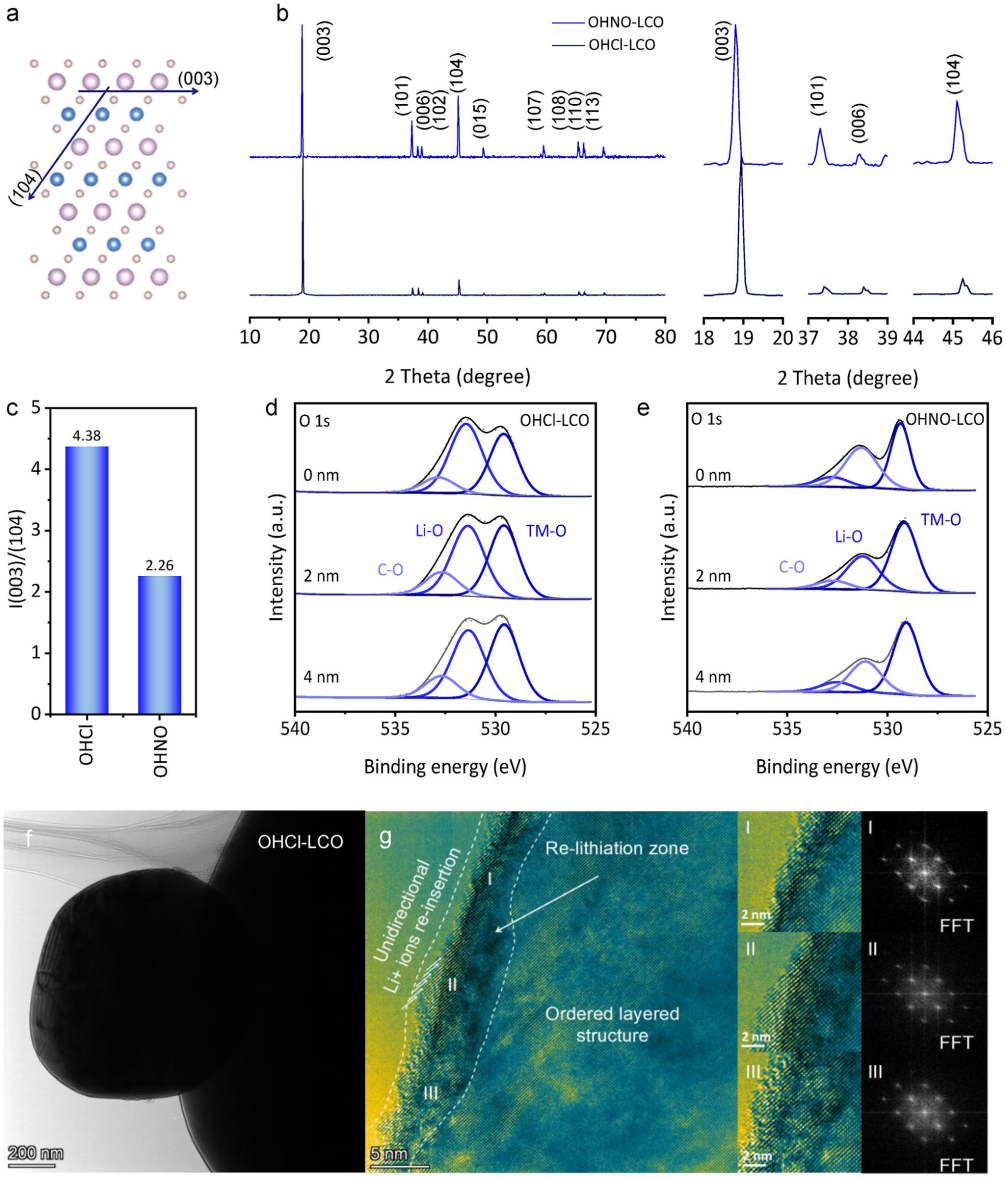
Cl^−^ anion enables directional Li^+^ ion reinsertion. (a) Schematic of the LCO (003) and (104) lattice planes. (b) XRD patterns and corresponding enlarged views of OHNO−LCO and OHCl−LCO. (c) Comparison of the diffraction peak intensity ratio I(003)/(104) of OHNO−LCO and OHCl−LCO. Depth profile of high‐resolution O 1s spectra of (d) OHNO−LCO and (e) OHCl−LCO. (f) TEM image of OHCl−LCO particle. (g) High‐resolution TEM image of OHCl−LCO particle with corresponding magnified images and FFT patterns from selected regions. The OHCl−LCO particle remain ordered layered structure at the bulk area and a obvious relithiaion zone on the near‐surface region wit distinct unidirectional Li^+^ ion reinsertion behavior.

To this end, according to the DFT results, two commonly binary eutectic systems (Figure S5, see supporting information for the detailed preparation processes of these two molten salts), LiOH−LiCl and LiOH−LiNO_3_, were selected to regenerate the spent LCO, producing LCO materials denoted as OHCl−LCO and OHNO−LCO, respectively. In the presence of highly concentrated molten lithium salts, the structural defects and lithium deficiency of spent LCO could be effectively recovered, resulting in the successful regeneration of the LCO cathodes in both systems (Figure 3a,b). Morphology evolution of spent LCO particles in these two eutectic systems provide clear reaction pathway in liquid‐solid relithiation system and offers physical evidence for the anion‐mediated mechanism proposed in our work (Figure S6 and Note S2). The Cl^−^ anion, which the DFT calculations show has a strong affinity for the LCO surface, not only directs the internal crystallographic reconstruction but also modifies the interfacial energy and mass transport kinetics at the particle surface. This enhances surface diffusion and promotes the Ostwald ripening behavior witnessed in OHCl−LCO.

Both OHCl‐LCO and OHNO‐LCO samples exhibit strong diffraction peaks without other impurities, indicating the successful elimination of spinel‐like components after the liquid−solid relithiation processes. Also, the well‐defined doublets (108)/(110) demonstrate the recovered layer structure [18]. Interestingly, different preferred orientations of the rebuilt lattice plane of recycled materials were observed (Enlarged sections in Figure 3b). Specifically, OHCl−LCO displays a significantly higher intensity ratio of (003) to (104) at 4.38, compared to OHNO−LCO, which has a ratio of 2.26, as shown in Figure 3c. This interesting observation implies that the short‐range‐order structure of coordinated Li^+^ ions in LiOH−LiCl and LiOH−LiNO_3_ contribute to distinct interactions on the surface of spent LCO, which affects the Li^+^ ion reinsertion process and the resulting preferred orientation of the reconstructed crystal plane.

To quantitatively address the crystal orientation changes, systematic Rietveld refinement was conducted and the March‐Dollase model was employed to refine the preferred orientation along the (003) plane. OHCl‐LCO sample exhibits a ratio of 0.768, higher than that of OHNO‐LCO (Figures S7, S8 and Table S1). Given that a value closer to unity indicates a more random crystallite orientation, these results quantitatively confirm the (003) preferred orientation in OHCl‐LCO. XPS depth profiling provides further evidence of the interactions and revealed a distinct anion‐derived surface environment of relevant LCO samples. Specifically, the depth profile of O 1s spectrum for OHCl−LCO (Figure 3d) shows a gradual change in the ratio of lattice oxygen to adsorbed oxygen species. More importantly, the peak assigned to adsorbed oxygen species, i.e., Li‐O species, of OHCl‐LiCl is consistently pronounced compared to OHNO−LCO (Figure 3e). These results indicate that LiOH‐LiCl binary molten salt system leads to a Li enrichment that involves a subsurface distribution. This could be attributed to the increased surficial Li−O species derived from the strong adsorption and fast dissociation of Li−Cl that leave more Li^+^ ion on the defective surface.

TEM imaging of OHCl−LCO (Figure 3f) provided direct visual evidence of the anion‐directed microstructural reconstruction. While both OHCl−LCO and OHNO−LCO particles exhibited a well‐ordered layered structure in the bulk, a distinct ∼7 nm relithiation zone was observed at the near‐surface region (Figure 3g, Figures S9‐S11). And the critical difference lies in the crystallographic orientation of this rebuilt interface. In OHCl−LCO, the relithiation front advanced in a remarkably uniform and preferred orientation manner (Figure 3g and Figure S9). High‐resolution imaging and the corresponding FFT patterns from multiple particles and regions (e.g., Regions I−III in Figure 3g, Figures S9 and S11) consistently revealed a single set of diffraction spots, exclusively indexed to the (010) and (100) planes of the layered phase. This demonstrates a highly consistent crystallographic orientation, indicative of epitaxial regrowth. In stark contrast, the relithiated surface of OHNO−LCO displayed anisotropic and random crystal orientations, as evidenced by a variety of FFT patterns corresponding to planes such as (101), (−102), and (015) (Figures S10, S11, and S13). The preferred directional Li^+^ reinsertion in OHCl−LCO, a finding robustly supported by detailed FFT and Inverse Fast Fourier Transform (IFFT) analysis across different particles (see Note S3 and Figures S9−S14), we believe, could be a direct consequence of the strong, specific interaction of Cl^−^ anions with the (010) plane, which templates the preferential lattice reconstruction along the *c*‐axis.

Yet the electrochemical performance of LCO is not positively correlated with the high I(003)/I(104) ratio. In fact, the high intensity of the (003) plane has been reported to be associated with inferior Li^+^ diffusion [30]. According to the tetrahedral site hop mechanism [31], Li migrates between the octahedral sites by passing through intermediate tetrahedral sites, and different facets of LCO exhibit varying Li^+^ diffusion activity. The (003) and (104) facets, being the facets with the lowest formation energy, are preferentially formed [32]. Whereas, it has been demonstrated that the (104) plane exhibits faster Li^+^ diffusion kinetics compared to the (003) plane, leading to a negative correlation between Li^+^ migration kinetics and the I(003)/I(104) ratio. Cyclic voltammetry (CV) test results (Figure S15) showed that the preferred orientation of reconstructed planes has limited effects on the reversibility of the cathodic and anodic reactions, which can be easily understood as the bulk structure of these materials remains the same. OHCl−LCO with a higher I(003)/I(104) ratio exhibited a relatively lower response current, which could be attributed to slower Li^+^ transfer kinetics in the (003)−dominated interface. The cycling performance of OHCl−LCO and OHNO−LCO was evaluated (Figures S16−S20). Although the capacity retention of OHCl−LCO (95%) is similar to that of OHNO−LCO (92%) (Figure S16), the latter experienced lower CE. Both cathodes shown obvious activation processes with low CE at the first several cycles (Figures S17 and S18). This could be facilely understood considering the relithiated Li^+^ ion on the surface. In terms of rate performance (Figure S19), within the voltage range of 3.0−4.2 V, OHCl−LCO displayed higher capacity at all rates. Moreover, OHCl−LCO electrode delivered better cycling stability and capacity retention at 1C (Figure S20). The higher deliverable capacity of OHCl−LCO could be ascribed to the maximized Li^+^ insertion within a limited time span, resulting from the strong interaction between Cl^−^ and the (010) plane. For desirable recycling or upcycling of spent LCO, achieving higher capacity and improved stability are the ultimate goals. Therefore, to enhance the kinetics of OHCl−LCO with a high I(003)/I(104) ratio, the exposed facet on the surface was further regulated through short annealing [33].

### 2.4 Enhanced Electrochemical Performance With Li‐O Enriched Surface

**FIGURE 4:**
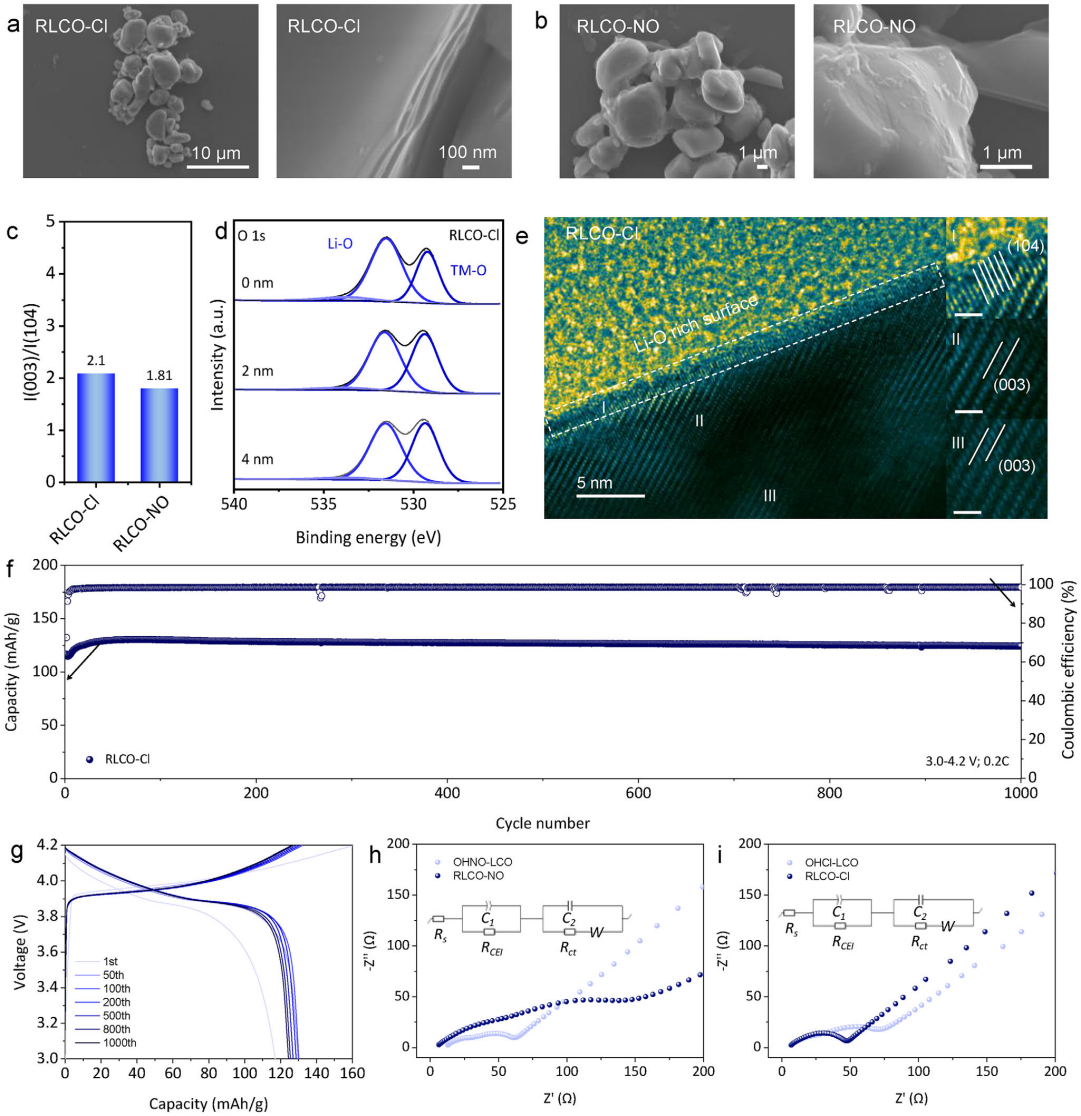
Directional relithiation induced Li−O enriched surface and enhanced cycling performance. SEM images of (a) RLCO−Cl and (b) RLCO−NO with magnified images. (c) Comparison of the I(003)/(104) ratio for RLCO−Cl and RLCO−NO. (d) Depth profiling of O 1s spectra of RLCO−Cl. (e) High‐resolution TEM image of RLCO−Cl with enlarged sections from selected regions in e. (f) Long−term cycling performance of RLCO−Cl at 0.2 C. (g) Corresponding charge−discharge voltage profiles of RLCO−Cl at various cycles. (h, i) Nyquist plots of OHNO−LCO/RLCO−NO and OHCl−LCO/RLCO−Cl electrodes after cycling for 200 cycles, respectively.

The distinct Li^+^ reinsertion behavior observed on OHCl‐LCO was found imperative in affecting the surface microstructure and electrochemical performance of annealed samples. After short annealing, the obtained materials are termed RLCO−Cl and RLCO−NO, respectively. Both RLCO−Cl and RLCO−NO display smooth surface with particle size ranging several micrometers (Figure 4a,b). The narrower XRD peaks which enable the separation of originally weak (111) and (0‐14) peaks from adjacent (101) and (104) peaks demonstrate the enhanced crystallinity (Figures S21−S23) [34]. The I(003)/I(104) ratio of RLCO−Cl decreased from 4.38 to 2.10 (Figure 4c), indicating a reorientation that exposes more of the electrochemically active (104) plane. We anticipate this change to the regrowth of the *ab*−plane of the annealed OHCl−LCO, resulting in an about 50% reduction in the intensity of the (003) peak. Rietveld refinement results present in Figures S24, S25 and Table S1 further support the decreased (003) preferred orientation of RLCO‐Cl after annealing. Meanwhile, the refined lattice parameters (a, c, and unit‐cell volume) of annealed samples and pre‐annealed ones show no significant differences, indicating that annealing does not affect the long‐range bulk crystal structure.

Moreover, the Cl^−^ anion‐derived surface microstructure feature, namely the subsurface Li enrichment observed in OHCl−LCO was found preserved in RLCO−Cl after annealing (Figure 4d). Compared with the pre‐annealed state, the Li−O to TM−O ratios of RLCO−Cl across the subsurface area remains largely unchanged. The persistence of abundant Li−O species on the RLCO−Cl surface directly reflects the effective and extensive interaction between the molten Li salt and the spent LCO particles during relithiation, in clear contrast to the RLCO−NO (Figure S26). This distinction is well rationalized by the working mechanism of the molten salt relithiation process. In this process, the molten Li salt interacts with the defective surface of LCO particles toreplenish Li, yet no extra external Co ions is introduced. Within a limited time frame, the amount of Li^+^ either re−inserted into lattice or adsorbed onto the surface is largely governed by the the interaction strength between the molten salt and the particle surface. Owing to the strong interaction between Cl^−^ and the defective (010) surface, Li^+^ ions preferentially bond to the CoO_6_ slabs and then diffuse to the Li vacancy sites. This not only enables sufficient relithiation but also leads to the accumulation of excess Li at the surface. Following heat treatment, aside from the Li^+^ that successfully reinsert into *R−3m* lattice vacancies, the remaining excess Li^+^ ions are likely incorporated into Co sites near the grain surface. The surface microstructure of RLCO−Cl and RLCO−NO present different features. Unlike the (003)−dominate interface and near−surface microstructure of RLCO−NO (Figure S27), the interface of RLCO−Cl was found exclosed by (104) plane (Figure 4e). This might originate from the lower surface energy nature of the (104) facet compared with the (003) plane.^25^ Co^3+^ on the (104) facet coordinates with five oxygen atoms, forming a square pyramidal configuration with low spin. And in the case of Li excess environment, the missing Co─O bonds would accordingly tune the crystal field, forming more Li─O bonds at the sub‐surface region, which in line with the XPS depth profiling results [35].

This (104)−enclosed and Li−O enriched interface empowered RLCO−Cl exceptional cycling stability. The RLCO−Cl cathode delivered a high capacity of 124.4 mAh/g and retained over 95% of its capacity after 1000 cycles (Figure 4f), with slightly increased polarization (Figure 4g). An obvious activation process was observed within the first 15 cycles, characterized by a significant initial irreversible capacity loss of over 40 mAh/g, followed by a steady climb in reversible capacity to approximately 130 mAh/g. This phenomenon, while present in both OHCl−LCO and OHNO−LCO cells, was more pronounced in the former. The Li−O enriched interface may require repeated cycling to be fully penetrated by the electrolyte and for optimal Li^+^ ion transport pathways to be established. The evolution of electrochemical impedance provides critical insights into the interfacial kinetics. Before the final calcination step, the charge transfer resistance (R_ct_) and cathode−electrolyte interphase resistance (R_CEI_) of OHNO−LCO were lower than those of OHCl−LCO after cycling. However, after calcination, the situation is dramatically reversed. The RLCO−Cl sample, with its newly formed Li−O enrich and (104)−enclosed surface, exhibits significantly lower R_ct_ and R_CEI_ values compared to RLCO−NO even after 200 cycles (Figure 4h,i).

**FIGURE 5:**
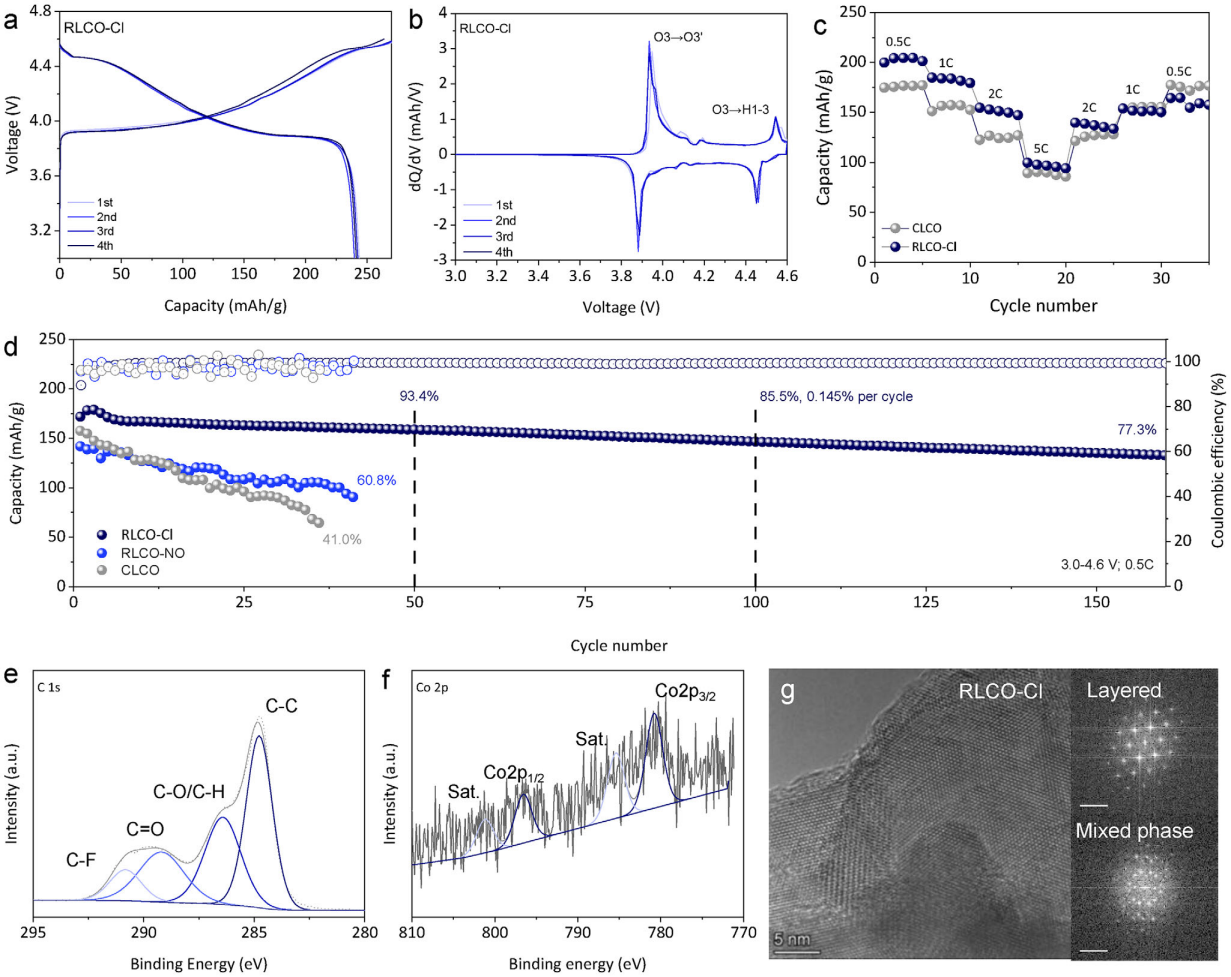
High−voltage cycling performance and post−cycling analysis. (a) Typical high‐voltage galvanostatic charge/discharge profiles of RLCO−Cl cathode. (b) Corresponding dQ/dV curves derived from a. (c) Rate capability comparison of RLCO−Cl and CLCO. (d) Discharge capacity against cycle number profiles of RLCO−Cl, RLCO−NO, and CLCO at 0.5 C within a voltage window of 3.0−4.6 V. High resolution (e) C 1s and (f) Co 2p XPS spectra of cycled RLCO−Cl cathode after cycling at 4.6 V. (g) Aberration−corrected TEM image of RLCO−Cl after cycling with FFT patterns from selected regions in g.

More importantly, the reconstructed interface of RLCO−Cl proved effective in enhancing high−voltage cycling performance using conventional carbonate electrolyte (1 M LiPF_6_ in EC:DEC). As shown in Figure 5a, RLCO−Cl delivers a high discharge capacity of 243.7 mAh/g at 0.1 C with well−defined plateaus. The corresponding dQ/dV curves confirm a highly reversible O3 to H1−3 phase transition, consistent with the CV results (Figure S28). This exceptional reversibility suggests the suppression of harmful phase separations, a common failure mode in high−voltage LCO. In contrast, RLCO−NO displayed much weaker redox peaks, indicating inferior electrochemical activity (Figure S29). Using the same electrolyte, commercial LCO (CLCO) without specific structural design for high‐voltage operation displays rather poor electrochemical performance (Figure 5c). Surprisingly, RLCO‐Cl, with it anion‐derived interface, retains 85.5% of its original capacity after 100 cycles at 0.5 C at 4.6 V (Figure 5d). Upon extended cycling to 160 cycles, the capacity retention remains at 77.3%. To further verify reproducibility, another cell assembled with RLCO‑Cl retained 81% and 72.8% capacity after 100 and 200 cycles, resepctively, with an averange CE of 99.35% (Figure S30). Compared with previously reported recycled LCO materials, RLCO‐Cl cathode in this work present desirable electrochemical performance using conventional electrolyte at both 4.2 and 4.6 V cut‐off voltages (Table S2). These electrochemical evaluation underscores the efficacy of rationally selecting anions to reconstruct the surface and subsurface structure during the regeneration process.

Post−cycling analysis confirmed the robustness of the RLCO−Cl interface (Figure 5e,f), yet due to the electrolyte decomposition, the thickness of CEI might be too thick for surface observation. While compared to its counterparts, clear Co 2p signal could be observed in RLCO−Cl after 100 cycles at 4.6 V (Figure S31). Aberration−corrected TEM (Figure 5g) revealed that the bulk layered structure remained largely intact after 100 cycles with mixed phase present at the near−surface region (Figure S32). This collective evidence demonstrates that the anion−guided relithiation play a vital role in reconstructing the interface microstructure that could alter the electrochemical performance. This work therefore establishes a crucial principle: the understanding and control of anion−mediated interface reconstruction might offer unique opportunities for upcycling high−voltage stable cathodes.

## 3 Conclusions

In conclusion, for the first time, the effects of different eutectic binary molten Li salts flux on the microstructure and electrochemical performance of recycled LCO cathode materials were investigated. The theoretical simulation and experimental results suggest that different molten salts feature varied effects on the reconstructed microstructure of the recycled materials, which originated from the anions−derived coordination environment of Li^+^. In the presence of Cl^−^, Li^+^ ions could interact strongly with the defective interfaces, which derives directional Li^+^ ion reinsertion and crystalline self−assembly due to the high adsorption energy to Li−Cl and suitable dissociation energy of Li─Cl bonds on the defective (010) plane of LCO. This interaction behavior leads to a directional crystallographic reconstruction of the spent LCO along the *c* axis, as represented by the ultrahigh I(003)/I(104) value of 4.38 for OHCl−LCO. The strong adsorption empowers sufficient Li^+^ re−insertion and the remaining surface bonded Li−O species, which was then shifted to Li enrichment on the surface of RLCO‐Cl with (104) as the main exposed facet. Benefiting from these features, the as−obtained RLCO‐Cl was successfully upcycled with better cycling performance at both 4.2 and 4.6 V. This work sheds light on the significance of anion interaction in recycling layered cathode materials in a liquid−solid reaction pathway.

## Author Contributions

M.Z., S.Z., and J.L. conceived the idea and designed the present work. M.Z. carried out the experiments and analyzed the data. M.L. performed the density functional theory calculations. S.Q. discussed and analyzed the data. M.Z., M.L., S.Z., and J.L. supported and supervised the study. M.Z. and J.L. wrote the manuscript. All authors discussed the results and commented on the manuscript.

## Funding

This work was supported by the China Postdoctoral Science Foundation (2025M770221 and 2025T180055). This work also acknowledges the support from National Natural Science Foundation of China (22509180, 2250090229, 22250710135, 22479030, 22579032).

## Conflicts of Interest

The authors declare no conflicts of interest.

## Supporting information




**Supporting File**: advs77656‐sup‐0001‐SuppMat.doc.

## Data Availability

The data that support the findings of this study are available from the corresponding author upon reasonable request.
