# Supplementary material for: Anion‐Driven Surface Reconstruction Enables Direct Regeneration of LiCoO2 Cathode Material
Source: Adv Sci (Weinh). 2026 Sep 27:e77656. Online ahead of print. doi: 10.1002/advs.77656 (PMC13616404; doi:10.1002/advs.77656)
Supplement: Supplementary file 1 [file ADVS-9999-e77656-s001.doc]

**Supporting information**

**Anion-Driven surface reconstruction enables direct regeneration of LiCoO_2_ cathode material**

Mengting Zheng^1,2^, Shangshu Qian^1,2^, Meng Li^3,^*, Shanqing Zhang^2,3^ and Jun Lu^1,^*

^1^ College of Chemical and Biological Engineering, Zhejiang University, Hangzhou 310027, China

^2^ Centre for Clean Environment and Energy, School of Environment and Science, Gold Coast Campus, Griffith University, Gold Coast 4222, Australia

^3^ Institute for Sustainable Transformation, School of Chemical Engineering and Light Industry, Guangdong University of Technology, Guangzhou 510006, China

*Corresponding author. E−mail:limeng@gdut.edu.cn; junzoelu@zju.edu.cn

**Materials and Methods**

**Figure S1 to S32**

**Supplementary Note 1 to 3**

**Table S1 to S2**

**Reference in supporting information**

**Materials and Methods**

**Harvest the spent LCO cathode materials.** Spent LCO prismatic batteries (Apple Co., Ltd.) were disassembled in the glove box and the obtained jelly rolls of the spent batteries were unrolled. The spent cathode layers were taken out and subjected to 550°C for 2 hours to remove the remaining electrolytes, binders, and conductive carbon. The remaining black powder substance was separated from the Al current collector layers and collected as the spent LCO cathode materials for subsequent processes.

**Regeneration of spent LCO materials.** The liquid−solid reactions happen in the eutectic molten salt system. In this work, two binary eutectic molten salt systems were investigated, including LiOH−LiCl and LiOH−LiNO_3_. The eutectic molten salt system is formed by adjusting the molar ratio between two lithium salts and the heating temperature. The phase diagrams of these two eutectic molten salts are presented in **Figure S5** and the blue dots in the figure represent the selected molar ratio of lithium salts and the corresponding eutectic molten temperature. In detail, for the preparation of LiOH−LiCl molten salt, the molar ratio of LiOH to LiCl is adjusted to be 3:2 with a eutectic temperature of about 340°C. LiNO_3_ and LiCl with a molar ratio of 3 to 2 were well mixed and then heated to 350°C under ambient pressure for 20 min. Once the eutectic liquid Li flux was formed, a certain amount of spent LCO cathode was added. The mixture was heated at the eutectic temperature, i.e., 350°C under ambient pressure for 4 hours. After the relithiation process, the mixture was cooled down and washed with DI water three times to remove all the remaining lithium salts. The as−obtained solid was vacuum−dried material and denoted as OHCl−LCO. To obtain annealed OHCl−LCO (RLCO-Cl), the OHCl−LCO was mixed with a certain amount of Li_2_CO_3_ (2 wt%) and the mixture was applied to 700°C for 2 hours under Ar atmosphere. These processes were applied to prepare LiOH−LiNO_3_ molten salt system, OHNO−LCO, and RLCO-NO except that the molar ratio between LiOH and LiNO_3_ was controlled to be 3:2.

**Preparation of LCO electrode.** Acetylene black (Swiss TIMCAL company), Polyvinylidene fluoride, PVDF (Solvay Technology Co., Ltd.), aluminium foil, Al (Henan Pengxiang Yunda), and N−methyl pyrrolidone, NMP (Sigma−Aldrich) were purchased and used. The individual LCO sample was mixed with acetylene black and PVDF in a mass ratio of 80:10:10 in N−Methyl−2−pyrrolidone (NMP) solvent. The slurries were stirred for 20 min and then coated on Al foil. The resultant electrode was vaccum dried in oven and then cut into sheets with a diameter of 13 mm with an active material loading of about 5 mg/cm^2^.

**Characterization.** The crystal structure of electrodes was analysed through XRD (Bruker, Bruker−Axe, Germany) with scanning rate of 5° min^−1^ with filtered Cu Kα radiation. HRTEM (Talos−S, FEI American) was conducted to investigate the microstructure of related LCO materials. Special attention was placed on the interface lattice arrangement, with Fast−Fourier pattern, and inverse Fast−Fourier patterns, XPS (ThermoESCALAB250) measurement was applied to shed light on the surficial compositions of individual LCO materials. XPS depth profiling of LCO samples were conducted on Nexsa G2 XPS (Thermo Fisher Scientific; 0.2nm/s).

**Electrochemical characterization.** All electrochemical measurements of galvanostatic charge/discharge tests were performed on Neware multichannel battery testing system (Neware Technology Ltd., China). The electrochemical workstation (CHI 600, Shanghai Chen Hua Instrument Company) was used and electrochemical impedance spectroscopy (EIS) tests. For low−voltage cycling tests, the voltage range was set to 3.0−4.2 V for the LCO||Li cells, and for high−voltage cycling tests, the voltage range was set to 3.0−4.6 V. The charge−discharge and rate performance of LCO||Li coin cells were evaluated on Neware MIHW−200−160CH under different C rates (1C=274 mAh/g). Rate performance was recorded after activation at 0.2 C for 5 cycles. Cyclic voltammetry (CV) was carried out on CHI660E (Huacheng, Shanghai) with a scan rate of 0.1 mV/s.

**Figure S1.** a, Pristine crystal structure of the LiCoO_2_. b, Crystal structure of LiCoO_2_ with Li vacancies (Li_v_).

**Figure S2.** The calculated adsorption energy and dissociation energy of Li−Cl and Li−NO_3_ on the lithium defective (010) plane of LCO.

**Figure S3.** The process to harvest the spent LCO materials from the decommissioned LIBs from cellphone batteries.

**Supplementary Note 1. The collection of spent LCO cathode materials.** Spent LCO prismatic batteries (Apple Co., Ltd.) showing open circuit voltage of about 0.5 V were opened in the glove box (H_2_O<0.1 ppm and O_2_<0.1 ppm). The obtained jelly rolls of the spent batteries were unrolled. The spent cathode layers were washed with dimethyl carbonate (DMC) for three times and then dried at 80^o^C in the oven. After that, the washed cathode layers were heated at 550^o^C for 2 hours to remove the remaining Poly(vinylidene fluoride) binder (PVDF, thermal decomposition temperature in the range of 300−450^o^C), and conductive carbon. Due to the decomposition of PVDF binder and the surface passivation of Al foil (the formation of passivated Al_2_O_3_), the remaining black powder could be easily separated from the Al current collector. These black mass were collected, milled, and sieving to obtain the spent LCO cathode materials for subsequent processes. The microstructure and remaining capacity of spent LCO cathode material were evaluated. The spent LCO cathode material was mixed with PVDF and Acetylene black in a mass ratio of 80:10:10 in NMP solvent. The slurry was stirred for 20 min and then coated on Al foil. The resultant electrode was dried in oven and then cut into sheets with a diameter of 13 mm with an active material loading of about 5 mg/cm^2^.

**Figure S4.** Corresponding Fast−Fourier transformation (FFT) patterns of region I, II, and III from Figure 2c

**Figure S5.** Phase diagram of a, the LiOH−LiCl and b, the LiOH−LiNO_3_ molten salt system. The blue dots in the figures indicate the molar ratio selected for the binary molten salt system and the corresponding eutectic temperature. The eutectic molten salt system is formed by adjusting the molar ratio between two lithium salts and the heating temperature. In detail, for the preparation of LiOH−LiCl molten salt, the molar ratio of LiOH to LiCl is adjusted to be 3:2 with a eutectic temperature of about 340^o^C. For the LiOH−LiNO_3_ molten salt system, the molar ratio between LiOH and LiNO_3_ is controlled to be 3:2, leading to a eutectic temperature of 325^o^C. To avoid the impacts of the amount of anions in the system, these molar ratios are intentionally selected.

**Figure S6.** SEM images of spent LCO, OHCl−LCO, and OHNO−LCO particles.

**Supplementary Note 2. Morphological Evolution of LCO Particles During Molten Salt Relithiation**

SEM was employed to track the morphological evolution of the cathode particles before and after the liquid−solid relithiation process, providing insights into the structural recovery and underlying particle regrowth dynamics. The spent LCO particles, harvested from commercial prismatic cells, largely retain their monolithic, single−crystal morphology with well−defined facets (**Figure S6**). However, close inspection reveals a corroded surface topography characterized by micro−cracks and surface roughening, which are typical manifestations of mechanical strain and structural degradation induced by repeated lithium (de)intercalation during cycling.

Following the relithiation treatment in both LiOH−LiCl (OHCl−LCO) and LiOH−LiNO_3_ (OHNO−LCO) eutectic systems, a significant morphological recovery is observed. The surfaces of the regenerated particles become notably smoother, and the micro−cracks appear healed, while the primary particle size and overall morphology remain largely unchanged. This observation indicates that the relithiation process occurs primarily through a repair and regrowth mechanism rather than a dissolution−recrystallization that would drastically alter particle size. The molten salt medium effectively provides a high−fluidity environment that facilitates mass transport for surface reconstruction, effectively “healing” the crystallographic defects without breaking down the primary particles.

A critical distinction emerges between the two salt systems upon closer examination. In the OHCl−LCO material, the SEM images frequently show particles that are “attached” or “bridged” at their facets (enlarged view in **Figure S6**). This is a classic signature of particle coarsening via sintering or Ostwald ripening in the presence of a liquid phase. The observation visually confirms that the LiOH−LiCl flux promotes significant atomic/molecular transport between adjacent particles, leading to the formation of necks and stronger interparticle connections. In contrast, such sintering features are conspicuously absent in the OHNO−LCO samples. The particles remain discrete with clear boundaries, suggesting a different and potentially less effective interfacial interaction in the LiOH−LiNO_3_ system.

**Figure S7.** The XRD Rietveld refinement result of OHCl-LCO

**Figure S8.** The XRD Rietveld refinement result of OHNO-LCO

**Table S1.** Rietveld refinement results of different LCO samples.

| Sample | Space group | March-Dollase ratio | Unit cell | | | Atom | x | y | z | Frac. | Uiso |
| --- | --- | --- | --- | --- | --- | --- | --- | --- | --- | --- | --- |
|  |  |  | a  (Å) | c(Å) | Vol |  |  |  |  |  |  |
| OHCl-LCO | R-3m | 0.768 | 2.81 | 14.04 | 96.10 | O1 | 0 | 0 | 0.27172 | 1 | 0.06696 |
|  |  |  |  |  |  | Co1 | 0 | 0 | 0.00000 | 1.0137 | 0.06696 |
|  |  |  |  |  |  | Li1 | 0 | 0 | 0.50000 | 1 | 0.06696 |
| RLCO-Cl | R-3m | 0.942 | 2.81 | 14.03 | 96.03 | O1 | 0 | 0 | 0.25257 | 1 | 0.08347 |
|  |  |  |  |  |  | Co1 | 0 | 0 | 0.00000 | 0.9949 | 0.08347 |
|  |  |  |  |  |  | Li1 | 0 | 0 | 0.5000 | 1 | 0.08347 |
| OHNO-LCO | R-3m | 0.828 | 2.80 | 13.99 | 94.98 | O1 | 0 | 0 | 0.25666 | 1 | 0.05939 |
|  |  |  |  |  |  | Co1 | 0 | 0 | 0.00000 | 1.0253 | 0.05939 |
|  |  |  |  |  |  | Li1 | 0 | 0 | 0.50000 | 1 | 0.05939 |
| RLCO-NO | R-3m | 0.886 | 2.81 | 14.01 | 95.58 | O1 | 0 | 0 | 0.26135 | 1 | 0.06310 |
|  |  |  |  |  |  | Co1 | 0 | 0 | 0.00000 | 1.0686 | 0.06310 |
|  |  |  |  |  |  | Li1 | 0 | 0 | 0.50000 | 1 | 0.06310 |

**Figure S9.** High−resolution TEM images of another OHCl−LCO particle showing unidirectional Li+ ion reinsertion behavior. a, TEM image of OHCl−LCO. b, High−resolution TEM image of OHCl−LCO with corresponding magnified images and FFT patterns from selected regions.

**Figure S10.** High−resolution TEM images of OHNO−LCO particle showing Li+ reinsertion along random direction at the surface relithiaion zone. a, TEM image of OHNO−LCO. b, High−resolution TEM image of OHNO−LCO with corresponding magnified images and FFT patterns from selected regions.

**Figure S11.** High−resolution TEM images of another OHNO−LCO particle showing Li+ reinsertion along random direction at the surface relithiaion zone. a, TEM image of OHNO−LCO. b, High−resolution TEM image of OHNO−LCO with corresponding magnified images and FFT patterns from selected regions.

**Figure S12.** HRTEM images and detailed crystallographic information of OHCl−LCO paricles. Enlarged sections along the surface areas, namely region i, ii, iii, iv, and v, and the corresponding FFT and Inverse Fast Fourier Transform (IFFT) patterns.

**Figure S13.** HRTEM images and detailed crystallographic information of OHNO−LCO paricles. Enlarged sections along the surface areas, namely region i, ii, iii, iv, and v, and the corresponding FFT and IFFT patterns.

**Figure S14.** a, The enlarged view of the surface area of OHCl−LCO. b, FFT and c, IFFT of the selected area in a. d, The corresponding crystal structure illustration.

**Supplementary Note 3. Crystallographic Evidence for Anion−Directed Unidirectional Relithiation** This note details the crystallographic analysis supporting the claim of unidirectional Li⁺ reinsertion in OHCl−LCO, as presented in Figures **S9−S12**. The consistent crystallographic orientation in the relithiated zone of OHCl−LCO was rigorously confirmed through Fast Fourier Transform (FFT) and Inverse Fast Fourier Transform (IFFT) analysis of multiple particles. In all FFT patterns derived from selected regions of OHCl−LCO particles (**Figures S9** and **S12**), we observed a singular and consistent set of diffraction spots. These spots were universally indexed to the (010) and (100) planes of the layered *R−3m* structure, with a measured lattice spacing of 2.45 Å for the (010) plane (**Figure S14**). The consistency of this pattern across different regions and particles confirms a highly aligned, unidirectional crystal regrowth. This is fundamentally different from the behavior observed in OHNO−LCO. The FFT patterns from its relithiated surface (**Figure S10** and **S11**) revealed a variety of diffraction spots corresponding to multiple lattice planes, such as (101), (−102), and (015). This diversity indicates a polycrystalline or randomly oriented reconstruction at the interface, lacking the templating effect found with Cl⁻.

To further isolate and visualize the specific planes responsible for the observed regrowth, IFFT filtering was employed on another OHCl−LCO particle (**Figure S14a**). By applying a mask to the (010) and (100) spots in the FFT (**Figure S14b**), the IFFT process reconstructs a real−space image containing only the information from these planes. The resulting IFFT image (**Figure S14c**) provides an unambiguous visualization of the perfectly aligned (010) and (100) lattice fringes at the OHCl−LCO surface. Since the (010) plane is vertical to the (001) plane, the accumulation and epitaxial growth along these directions directly facilitate the preferential regrowth of the crystal along the *c*−axis (**Figure S14d**).

In conclusion, this comprehensive crystallographic analysis demonstrates that Cl⁻ anions direct a specific, preferred lattice reconstruction by templating growth along the (010) and (100) planes. In contrast, NO_3_^−^ anions, with weaker and less specific adsorption, lead to a multidirectional and disordered reconstruction. These findings provide the microstructural foundation for the anion−mediated mechanism governing crystal regeneration in molten salts.

**Figure S15.** Cyclic voltammetry of a, the OHCl−LCO and b, the OHNO−LCO cells at scan rate of 0.1 mV/s.

**Figure S16.** Cycling performance of the OHNO−LCO and OHCl−LCO cells at 0.2 C in a voltage range of 3.0−4.2 V.

**Figure S17.** The charge−discharge voltage profiles of the OHCl−LCO cells at different cycles at 0.2 C in a voltage range of 3.0−4.2 V.

**Figure S18.** The charge−discharge voltage profiles of the OHNO−LCO cells at different cycles at 0.2 C in a voltage range of 3.0−4.2 V.

**Figure S19.** The rate performance of OHCl−LCO and OHNO−LCO at 0.1C, 0.2C, 0.5C, and 1C.

**Figure S20.** The cycling performance f OHCl−LCO and OHNO−LCO at 1C in a voltage range of 3.0−4.2 V.

**Figure S21.** XRD patterns of RLCO−NO and RLCO−Cl, with the enlarged section showing peaks at the range of 18−19^o^, 37−39^o^, and 44−46^o^.

**Figure S22.** HRTEM image of RLCO−Cl and the corresponding selected area electron diffraction (SAED) pattern.

**Figure S23.** HRTEM image of RLCO−NO and the corresponding SAED pattern.

**Figure S24.** The XRD Rietveld refinement result of RLCO-Cl.

**Figure S25.** The XRD Rietveld refinement result of RLCO-NO.

**Figure S26.** XPS sputtering of O 1s spectra of RLCO−NO.

**Figure S27.** Magnified HRTEM image of the surface region of RLCO−NO material.

**Figure S28.** Cyclic voltammetry curves of RLCO−Cl electrode at a scan rate of 0.1 mV/s.

**Figure S29.** Cyclic voltammetry curves of RLCO−NO electrode at a scan rate of 0.1 mV/s.

**Figure S30.** Discharge capacity against cycle number profiles of another cell using RLCO−Cl cathode at 0.5 C within a voltage window of 3.0−4.6 V.

**Table S2.** Comparison of electrochemical performance between RLCO‑Cl and previously reported recycled LCO cathodes at different cut‑off voltages and rates.

| **Methods** | **Electrolyte** | **Discharge capacity**  **(mAh/g)** | **Cycling numbers** | **Capacity retention**  **(%)** | **Voltage**  **(V)** | **Ref.** |
| --- | --- | --- | --- | --- | --- | --- |
| Molten salt regeneration | LiPF_6_ in EC/DMC/EMC | 147.3 (0.2C) | 100 | 93 (0.2C) | 4.25 | ^[1]^ |
| DES regeneration | LiPF_6_ in EC/DMC | 130.0 (0.1C) | 100 | 90 (0.5C) | 4.2 | ^[2]^ |
| Anion-derived liquid-solid relithiaion | LiPF_6_ in EC/DEC | 130.0 (0.2C) | 1000 | 95 | 4.2 | This work |
| Solid-state sintering | LiPF_6_ in EC/DMC/DEC | 220.4 (0.1C) | 300 | 79.7 (0.5C) | 4.6 | ^[3]^ |
| Ball milling+ ultrafast repaired method | LiPF_6_ in EC/DMC/EMC | 133.0 (0.1C) | 300 | / | 4.3 | ^[4]^ |
| Solid-state sintering | LiPF_6_ in EC/DMC/DEC | 175.2 (1C) | 500 | 88.9 (5C) | 4.5 | ^[5]^ |
| Chemical re-lithiation | LiPF_6_ in EC/EMC/DMC | 141.8 (0.2C) | 400 | 76.8 (0.33) | 4.3 | ^[6]^ |
| Ca^2+^ assisted molten salt regeneration | LiPF_6_ in EC/DMC/DEC | 108.7 (4C) | 300 | 79.7 (2C) | 4.3 | ^[7]^ |
| Solid-state sintering | LiPF_6_ in EC/DEC | 148.3 (0.1C) | 25 | 80.0 (0.1C) | 4.2 | ^[8]^ |
| Solid-state sintering | LiPF_6_ in EC/DMC/DEC | 220.7 (0.1C) | 300 | 89.7 (10C) | 4.6 | ^[9]^ |
| Ball milling +coating | LiPF_6_ in FEC:HFE:FEMC | / | 250 | 90.1 (0.5C) | 4.6 | ^[10]^ |
| Ball milling | LiPF_6_ in EC/DMC/DEC | 134.7 (2C) | 200 | 92.0 (2C) | 4.3 | ^[11]^ |
| Solid-state sintering | LiPF_6_ in EC/DMC/DEC | 201.0 (0.2C) | 200 | 86.8 (3C) | 4.5 | ^[12]^ |
| Ball milling +doping | LiPF_6_ in EC/DMC/DEC | 227.8 (0.1C) | 300 | 85.7 (1C) | 4.6 | ^[13]^ |
| DES regeneration | LiPF_6_ in EC/DMC/EMC | 183.1 (0.2C) | 200 | 87.0 (0.5C) | 4.5 | ^[14]^ |
| Hydrothermal | LiPF_6_ in EC/DMC/DEC | 141.1 (1C) | 300 | 92.9 (1C) | 4.3 | ^[15]^ |
| Solid-state sintering +doping | LiPF_6_ in EC/DEC | 201.0 (1C) | 100 | 77.3 (1C) | 4.6 | ^[16]^ |
| Anion-derived liquid-solid relithiaion | LiPF_6_ in EC/DEC | 171.1 (0.5C) | 100 | 85.5 (0.5C) | 4.6 | This work |

**Figure S31.** High resolution C1s and Co 2p XPS spectra of cycled RLCO−NO cathode.

**Figure S32.** Aberration−corrected TEM and magnified images of RLCO−NO after cycling at 4.6 V for 100 cycles.

Reference in supporting information

[1]	H. Yang, B. Deng, X. Jing, W. Li, D. Wang, *Waste Manag.* **2021**, *129*, 85.

[2]	J. Wang, Q. Zhang, J. Sheng, Z. Liang, J. Ma, Y. Chen, G. Zhou, H.-M. Cheng, *Natl Sci Rev* **2022**, *9*, nwac097.

[3]	J. Wang, K. Jia, J. Ma, Z. Liang, Z. Zhuang, Y. Zhao, B. Li, G. Zhou, H.-M. Cheng, *Nat Sustain* **2023**, *6*, 797.

[4]	Y.-C. Yin, C. Li, X. Hu, D. Zuo, L. Yang, L. Zhou, J. Yang, J. Wan, *ACS Energy Lett.* **2023**, *8*, 3005.

[5]	H. Lei, Z. Zeng, J. Li, X. Cui, B. Wang, Y. Shi, W. Sun, X. Ji, Y. Yang, P. Ge, *Advanced Functional Materials* **2024**, *34*, 2402325.

[6]	M. Xu, C. Wu, L. Ye, Y. Zhang, C. Zhang, J. Hu, R. Tan, D. Gu, X. Wang, O. Fontaine, C. Zhan, L. Zhuang, X. Ai, J. Qian, *Advanced Energy Materials* **2024**, *14*, 2401197.

[7]	G. Gao, Y. Zhu, S. Di, J. Zhao, C. Liu, S. Wang, L. Li, *Acta Materialia* **2024**, *273*, 119969.

[8]	S. Kumar, K. Chakravarty, K. Kumar, A. Sen, C. Ghanty, S. Chakravarty, R. Kundu, *Energy Fuels* **2024**, *38*, 6499.

[9]	D. Fang, F. Wu, X. Zhang, X. Zhang, Y. Ye, R. Chen, L. Li, *Angewandte Chemie International Edition* **2025**, *64*, e202502176.

[10]	Y. Yan, D. Chen, Z. Sun, Q. Zheng, W. Li, J. Xue, Y. Chen, C. Wang, C. Wang, H. Liao, P. Zhang, C. Qu, J. Wang, Y. Sun, Q. Zhang, Y. Qiao, S. Sun, *Angew Chem Int Ed* **2025**, e202512300.

[11]	F. Zengin, S. Küçükköylü, M. N. Ateş, *ACS Sustainable Chem. Eng.* **2025**, *13*, 12372.

[12]	G. Gao, Y. Zhang, Y. Zhu, Z. Li, X. Tong, J. Choi, S. Wang, L. Li, *Energy Storage Materials* **2025**, *83*, 104734.

[13]	Q. Yan, Z. Liu, F. Wu, R. Chen, L. Li, *Advanced Materials* **2026**, *38*, e19347.

[14]	S. Zhang, Y. Liu, G. Chen, Z. M. El-Bahy, D. A. Alshammari, M. H. Helal, J. Zhou, J. Han, *Advanced Materials* **2026**, *38*, e13547.

[15]	G. Gao, Y. Zhu, Z. Li, C. Liu, J. Zeng, T. Liu, S. Wang, S. Chun, T. Yi, L. Li, *Acta Materialia* **2026**, *302*, 121663.

[16]	Y. Deng, H. Yang, G. Sun, Y. Dai, X. Jiang, Y. Chen, S. Qiao, Z. Wang, L. Li, L. Chen, Y. Zhang, W. Luo, *Sci. China Chem.* **2026**, *69*, 2544.
